# Isthmin-1 (ISM1), a novel adipokine that reflects abdominal adipose tissue distribution in individuals with obesity

**DOI:** 10.1186/s12933-023-02075-0

**Published:** 2023-12-08

**Authors:** Marta Lopez-Yus, Carmen Casamayor, Juan Jose Soriano-Godes, Sofia Borlan, Yolanda Gonzalez-Irazabal, Maria Pilar Garcia-Sobreviela, Beatriz Garcia-Rodriguez, Raquel del Moral-Bergos, Pilar Calmarza, Jose Maria Artigas, Silvia Lorente-Cebrian, Vanesa Bernal-Monterde, Alejandro Sanz-Paris, Jose M. Arbones-Mainar

**Affiliations:** 1 Adipocyte and Fat Biology Laboratory (AdipoFat), Translational Research Unit, University Hospital Miguel Servet, Instituto Aragones de Ciencias de la Salud (IACS), Zaragoza, Spain; 2 Instituto de Investigación Sanitaria (IIS) Aragon, Zaragoza, 50009 Spain; 3 Endocrine, Bariatric and Breast Surgery Unit, General and Digestive Surgery Department, Miguel Servet University Hospital, Zaragoza, Spain; 4 Department of Radiology, Royo-Villanova Hospital, Zaragoza, Spain; 5 Clinical Biochemistry Department, Miguel Servet University Hospital, Zaragoza, Spain; 6 CIBER Enfermedad Cardiovascular (CIBERCV), Instituto Salud Carlos III, Madrid, Spain; 7 Department of Radiology, Miguel Servet University Hospital, Zaragoza, Spain; 8 Department of Pharmacology, Physiology and Legal and Forensic Medicine, Faculty of Health and Sport Science, University of Zaragoza, Zaragoza, Spain; 9 Instituto Agroalimentario de Aragón (IA2) (Universidad de Zaragoza-CITA), Zaragoza, Spain; 10 Gastroenterology Department, Miguel Servet University Hospital, Zaragoza, Spain; 11 Endocrinology and Nutrition Department, Miguel Servet University Hospital, Zaragoza, Spain; 12 CIBER Fisiopatología Obesidad y Nutrición (CIBERObn), Instituto Salud Carlos III, Madrid, Spain; 13 Adipocyte and Fat Biology Laboratory (AdipoFat), Unidad de Investigación Traslacional, Hospital Universitario Miguel Servet, Instituto Aragonés de Ciencias de la Salud (IACS), Instituto de Investigación Sanitaria (IIS) Aragón, Isabel la Católica, 1-3, Zaragoza, 50009 Spain

**Keywords:** CT-Scan, Biomarker, Subcutaneous fat, Expandability

## Abstract

**Background:**

The assessment of obesity-related health risks has traditionally relied on the Body Mass Index and waist circumference, but their limitations have propelled the need for a more comprehensive approach. The differentiation between visceral (VIS) and subcutaneous (SC) fat provides a finer-grained understanding of these risks, yet practical assessment methods are lacking. We hypothesized that combining the SC-VIS fat ratio with non-invasive biomarkers could create a valuable tool for obesity-related risk assessment.

**Methods and results:**

A clinical study of 125 individuals with obesity revealed significant differences in abdominal fat distribution measured by CT-scan among genders and distinct models of obesity, including visceral, subcutaneous, and the SC/VIS ratio. Stratification based on these models highlighted various metabolic changes. The SC/VIS ratio emerged as an excellent metric to differentiate metabolic status. Gene expression analysis identified candidate biomarkers, with ISM1 showing promise. Subsequent validation demonstrated a correlation between ISM1 levels in SC and plasma, reinforcing its potential as a non-invasive biomarker for fat distribution. Serum adipokine levels also correlated with the SC/VIS ratio. The Receiver Operating Characteristic analysis revealed ISM1’s efficacy in discriminating individuals with favorable metabolic profiles based on adipose tissue distribution. Correlation analysis also suggested that ISM1 was involved in glucose regulation pathways.

**Conclusion:**

The study’s results support the hypothesis that the SC-VIS fat ratio and its derived non-invasive biomarkers can comprehensively assess obesity-related health risks. ISM1 could predict abdominal fat partitioning and be a potential biomarker for evaluating obesity-related health risks.

## Background

The Body Mass Index (BMI) and waist circumference have been widely used to assess obesity, but their limitations have prompted calls for a more comprehensive approach. BMI’s sole reliance on weight and height overlooks variations in body composition, potentially misclassifying muscular individuals as obese. Similarly, while considering fat distribution, waist circumference, and waist/hip ratio can be limited as they cannot adequately differentiate between subcutaneous and visceral fat, which is crucial for assessing obesity-related health risks.

White visceral adipose tissue (visWAT), located within the abdominal cavity and intertwined with vital organs, is associated with cardiovascular disease, type 2 diabetes (T2DM), hypertension, and fatty liver [1–3]. It can release pro-inflammatory mediators, contributing to these health risks [4]. In contrast, subcutaneous fat (scWAT), found just beneath the skin’s surface, while still contributing to overall body fat, is less metabolically active and does not pose a metabolic threat [5–7]. This differentiation between visceral and subcutaneous fat provides a finer-grained understanding of an individual’s obesity-related health risks. It is hence of paramount importance the evaluation of the quantity and distribution of fat in the body. Consequently, focusing on this ratio would be valuable in identifying individuals at higher risk and tailoring interventions to lower morbidity and mortality rates.

However, the challenge lies in implementing this assessment. Imaging techniques, such as magnetic resonance imaging (MRI), computed tomography (CT), and dual-energy x-ray absorptiometry (DXA), provide accurate results but are often complex and impractical in many clinical settings. In this context, non-invasive biomarkers come into play. These indicators, like specific adipokines or hormonal markers, can be measured in blood or urine samples. By measuring these biomarkers, clinicians could estimate the subcutaneous-visceral fat ratio without the need for sophisticated analysis.

Adipokines such as adiponectin and leptin have been associated with fat distribution patterns and metabolic health [8, 9]. However, the current knowledge of these adipokine concentration variations is still unable to explain the heterogeneity of metabolic risk. Therefore, there is still a need to identify a promising biomarker that appropriately reflects the adipose tissue distribution and, consequently, the metabolic status of an individual.

We hypothesize that by combining the concept of the subcutaneous-visceral fat ratio with non-invasive biomarkers, we can create a powerful tool to assess obesity-related health risks comprehensively and practically. This approach would enable healthcare professionals to identify at-risk individuals more effectively, allowing for timely interventions and enabling personalized medicine in obesity. Accordingly, the objectives of this study are twofold: We aim to (1) investigate the relationship between abdominal adipose components (scWAT and visWAT) and metabolic dysfunction associated with obesity and (2) identify non-invasive biomarkers associated with abdominal adipose distribution that do not require the use of advanced imaging techniques.

## Materials and methods

### Human samples and cohort description

Individuals with obesity were recruited at either the Miguel Servet University Hospital (HUMS, Zaragoza, Spain) or the Royo-Villanova Hospital (HRV, Zaragoza, Spain) as described previously [10]. Briefly, all patients scheduled for elective bariatric surgery were offered the opportunity to participate in the study by donating blood, biopsies of subcutaneous and visceral adipose tissue, and undergoing a computerized tomography (CT) scan for the quantitative determination of subcutaneous and visceral abdominal fat depots. All patients provided written consent and the study was approved by the Regional Institutional Review Board of Ethics at Aragón, Spain (CEIC-A). Samples and data from patients included in this study were provided following standard operating procedures by the Biobank of the Aragon Health System (PT20/00112), integrated into the Spanish National Biobanks Network.

### Determination of abdominal fat distribution

The visceral and subcutaneous fat areas were measured by CT with an 8 mm single slice at the umbilical level. All CT examinations were acquired with the subject positioned supine in a 64 detector CT scanner (Aquilion 64 Toshiba, Japan) and tube voltage set to 120 kVp with automatic tube current modulation and rotation time of 0.5 s. Acquired images were then transferred to a workstation and analyzed with the Vitrea CT Fat Measurement software (Vital Imaging Inc. The Netherlands). Selected fat densities ranged between − 150 and − 70 Hounsfield Units (HU) and the areas of the total subcutaneous (SFA) and visceral fat (SFA) were measured in cm^2^. SFA and VFA were respectively defined as pixels (area) located outside or inside the outer surface of the abdominal muscle wall. Subsequently, we calculated the ratio between subcutaneous fat area divided by the area of visceral fat area (SFA/VFA ratio).

### Biochemical assays

C-reactive protein (CRP), glycated hemoglobin (Hb1Ac), cross-linked C-terminal telopeptide of type I collagen (CTx), triglyceride (TG), cholesterol associated with high-density lipoproteins (HDLc), non-esterified fatty acids (NEFA), gamma glutamil transferase (GGT), alanine transaminase (ALT), and leptin were determined at the Clinical Biochemistry department at the HUMS using state of the art analyzers. All analyses were in compliance with the requirements for quality and competence (ISO 15189:2012) for medical laboratories. Homeostatic Model Assessment for Insulin Resistance (HOMA) was calculated as previously described [11].

Adiponectin, adipsin, MCP-1 and resistin were measured using the LegendPlex immunoassay (Biolegend) by flow cytometry, following manufacturer’s instructions. The process was carried out in the Cell Sorting and Cytometry Unit of the Aragonese Institute of Health Sciences (IACS). ISM1 and CCDC3 were determined by FineTest ELISA kits (refs. EH4520 and EH0082, respectively) according to the manufacturer’s instructions (Fine Biotech Co.)

### RNA isolation

Total RNA was isolated from frozen biopsies of scWAT and visWAT using TRIzol (Sigma) according to the manufacturer’s protocol. All the RNA samples were treated with RNase-Free DNase (Life Technologies) to remove genomic DNA.

### AmpliSeq human transcriptome analysis

RNA content was measured using the Qubit RNA high-sensitivity (HS) assay kit (#Q32855, ThermoFisher) with the Qubit 3.0 fluorometer (ThermoFisher). RNA integrity was checked by the 2200 TapeStation system (Agilent) with the High Sensitivity RNA ScreenTape (5067–5579, Agilent). All samples had an RNA integrity number (RIN) > 7. RNA (20 ng) was converted to cDNA using the SuperScript IV VILO Master Mix (#11,756,050, ThermoFisher) and libraries were made with the Ion AmpliSeq transcriptome human gene expression panel (#A31446, ThermoFisher) on the Ion Chef Prep station(ThermoFisher).

Sequencing was performed using the Ion 540 Kit-Chef (# A30011, ThermoFisher) on the Ion Torrent S5-XL (ThermoFisher). Eight samples *per* 540 chip were sequenced, obtaining 7.5 to 10 million reads/sample. The entire sequencing process was carried out in the Genomics Unit of the Aragonese Institute of Health Sciences (IACS).

Next, bam files were processed using the Ion AmpliSeq RNA plugin v5.4.01 on the Torrent Server (Torrent Suite Software, v5.4, ThermoFisher) to obtain to obtain the count matrices. Differential expression analysis was performed with the edgeR R package (v. 3.40.02) [12]. The gene expressions of the scWAT samples included in the first tertile were compared to the scWAT samples included in the third tertile. Briefly, samples were normalized using a weighted trimmed mean of M-values (TMM) and fitted to a quasi-likelihood negative binomial generalized log-linear model followed by empirical Bayes quasi-likelihood F-tests. Genes with log2 fold changes |log2(FC)| ≥ 1 and Benjamini and Hochberg false discovery rates (FDR, [13]) < 0.1 were classified as differentially expressed genes (DEGs).

### RT-qPCR

RNA was reverse-transcribed using PrimeScript Reverse Transcriptase (Takara Bio). Real-time PCR was performed using the StepOnePlus system (Applied Biosystems). 2 µl of the cDNA product was amplified using gene-specific primers in a total volume of 15 µl *per* reaction with SYBR Select Master Mix (Applied Biosystems). Relative gene expression was normalized to *β-ACTIN* expression using the 2^−△△Ct^ method. The following primers were used: *ISM1*-F, 5’-CTTCCCCAGACCGCGATTC-3’; *ISM1*-R, 5’-CGACCACCTCTATGGTGACCT-3’; *CCDC3*-F, 5’-TGACTGGGAAATCCAGGAAGA-3’; *CCDC3*-R, 5’-CGTGGTCCTCCTCCTCAAAC-3’; *ACTIN*-F, 5’-CATGTACGTTGCTATCCAGGC-3’; *ACTIN*-R, 5’-CTCCTTAATGTCACGCACGAT-3’.

### Western blot analysis

For analysis of protein expression, aliquots of serum or adipose tissue biopsies lysates were subjected to reducing sodium dodecyl sulfate-polyacrylamide gel electrophoresis (SDS-PAGE), using 12% polyacrylamide gels. Electrophoresis was carried out at 100 volts for 2 h. Proteins were then transferred to polyvinylidene difluoride membranes (PVDF), which were blocked with 5% dry skimmed milk for 2 h and probed with primary antibody against ISM1 or β-ACTIN overnight at 4 ºC with mild shaking. The results were visualized using fluorophore–conjugated secondary antibodies that were incubated 1 h at room temperature with mild shaking. Antibody used were: β-Actin (Santa Cruz #SC-47,778) diluted 1:1000, ISM1 (Thermo Fisher #PA5-24968) diluted 1:1000, mouse secondary antibody (Thermo Fisher # A-11,001) diluted 1:30000, rabbit secondary antibody (Thermo Fisher # A-21,206) diluted 1:30000.

### Statistical analysis

Boxplots were used to display the distribution and summary statistics. The line within the box indicates the median value, the box represents the interquartile range (IQR), and the whiskers extend from the edges of the box to the minimum and maximum values within 1.5 times the IQR. Data points beyond this range are plotted individually. Spearman correlation analysis was used to investigate the association between continuous variables. We calculated *p*-values for trends in ordered categories (tertiles) using the Pearson or the Spearman tests for normal- or non normal-distributed variables, respectively. We utilized Receiver Operating Characteristic (ROC) curves to assess the predictive power and discriminative ability of our classification model. We quantified the overall performance of our model by calculating the area under the ROC curve (AUC). The statistical analysis was performed using R version 4.0.3 (http://www.r-project.org) and the appropriate packages, *p*-values < 0.05 were considered significant.

## Results

### Metabolic characterization of the different subtypes of obesity

This clinical study included 125 individuals with obesity. The median age was 49 yrs (range 20–66) and 57% were women. The median BMI and waist circumference were 42 kg/m^2^ and 120 cm, respectively, with no observed sex-based differences in these parameters (as shown in Table 1). All participants underwent a CT-scan imaging test to determine abdominal fat distribution, measuring Subcutaneous Fat Area (SFA) and Visceral Fat Area (VFA). Significant disparities emerged in adipose partition, with women having higher subcutaneous fat and lower visceral adipose tissue than men.

**Table 1:** Clinical and biochemical characteristics of the subjects in the cohort

	[ALL]	Men	Women	*p*
	*N = 125*	* N = 54*	* N = 71*	
Age (years)	49.0 [41.0;56.0]	48.5 [45.0;58.0]	49.0 [40.0;54.0]	0.308
Body mass index (BMI, kg/m^2^)	41.0 [35.9;44.6]	40.4 [35.9;44.5]	41.0 [36.3;44.5]	0.884
Waist circumference (cm)	120 [112;126]	122 [114;130]	120 [112;125]	0.335
Subcutaneous Fat Area (cm^2^)	355 [248;478]	270 [205;375]	403 [310;494]	< 0.001
Visceral Fat Area (cm^2^)	239 [154;335]	332 [268;418]	190 [121;250]	< 0.001

Data are median [interquartile range]

*p*: *p*-value for the sex differences (Mann-Whitney)

**Fig. 1:**
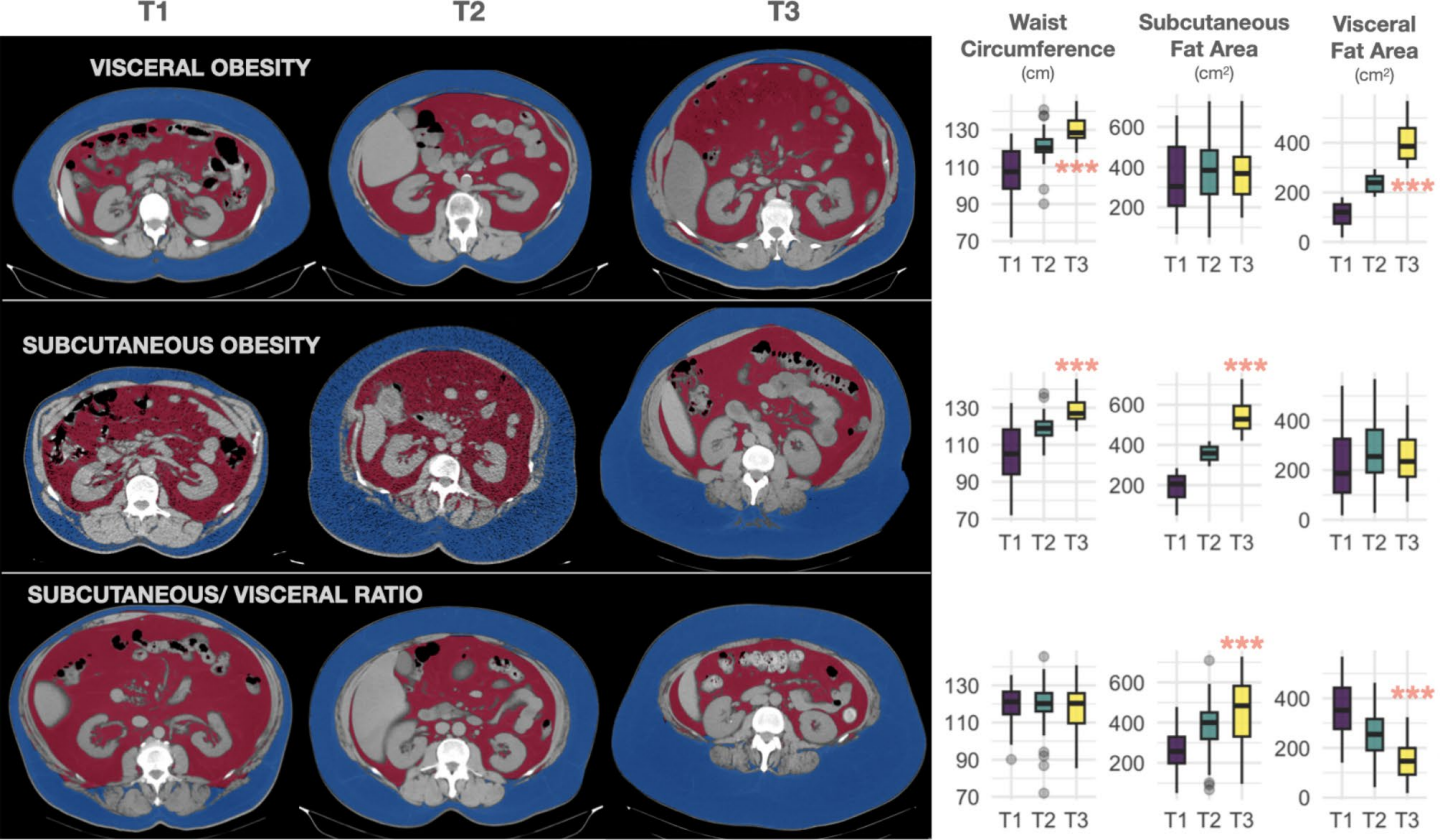
Representative CT scan of abdominal adipose tissue segmentation for each group of people with obesity, according to visceral (**A**), subcutaneous adipose tissue accumulation (**B**) or subcutaneous/visceral ratio (**C**). Subcutaneous and visceral fat are colored in blue and red, respectively T1 represented the lowest tertile while T3 represented the highest tertile of VF area, SCF area, and SCF/VF areas, respectively

We categorized obesity into three distinct models based on the enlargement of adipose deposits: 1)Visceral obesity (classical android obesity) characterized by increased VFA and constant SFA, 2) Subcutaneous obesity (classical gynoid pattern) characterized by enlarged SFA and constant VFA, and 3) the SFA/VFA ratios, utilized to assess the propensity for visceral versus subcutaneous fat storage. We further divided these models into tertiles to facilitate comparisons among them. Notably, the first two types of obesity exhibited an increase in waist circumference across obesity tertiles, whereas the third type maintained a consistent waist circumference across tertiles (Fig. 1).

**Fig. 2:**
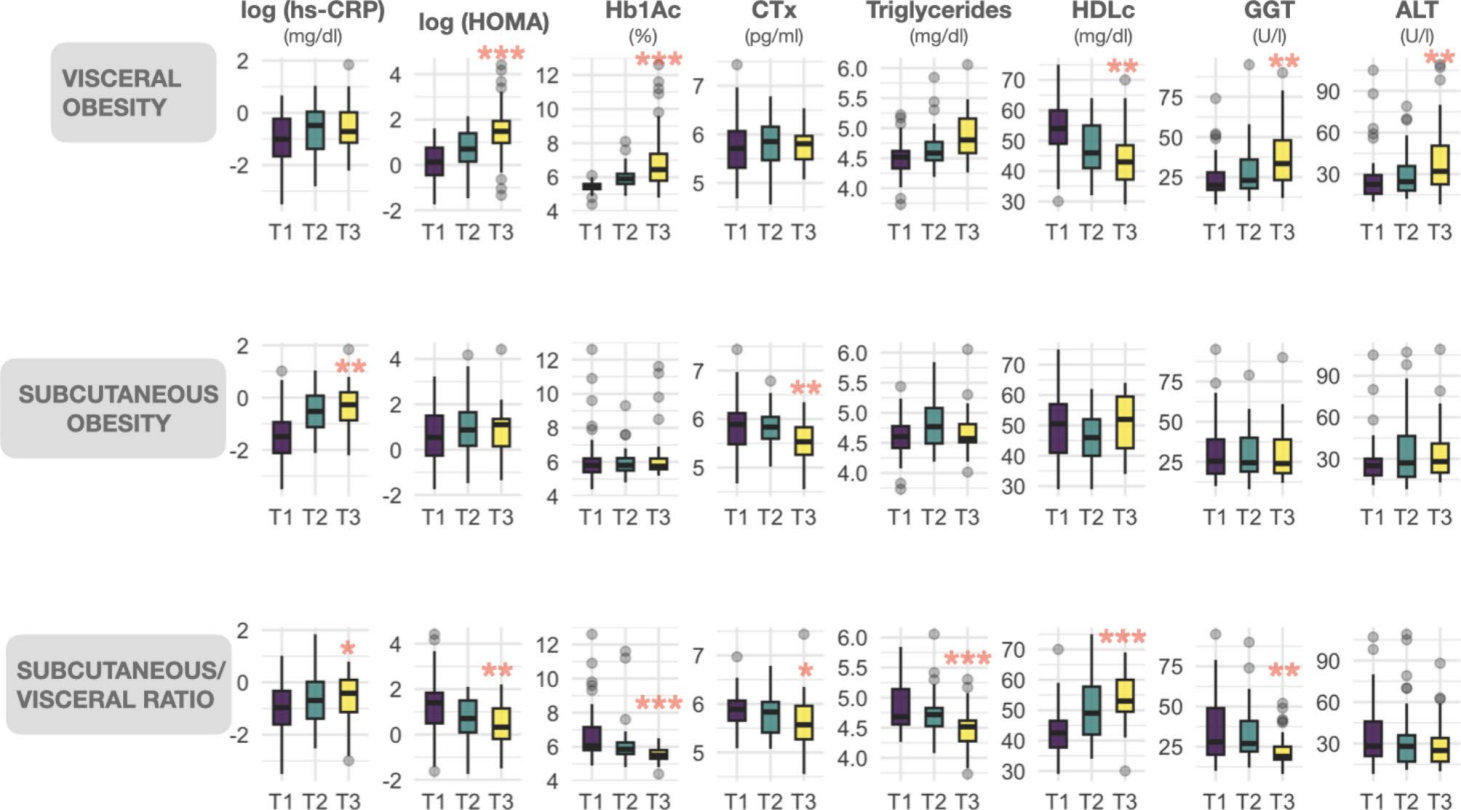
Morphometric and metabolic parameters in the lower, middle and upper tertiles of visceral and subcutaneous adipose tissue accumulation and subcutaneous/visceral ratio in people with obesity Hs-CRP: high-sensitivity C-reactive protein, HOMA: homeostasis model assessment of insulin resistance, Hb1Ac: glycated hemoglobin, Ctx: cross-linked C-terminal telopeptide of type I collagen, TG: plasma triglycerides, HDLc: cholesterol associated with high density lipoproteins, GGT: gamma glutamil transferase, ALT: alanine transaminase. p for trend across tertiles: *<0.05, **<0.01,***<0.001

Clinical and biochemical parameters were measured and analyzed according to the different classifications of obesity (Fig. 2). Upon stratification based on visceral obesity, we noted a compromised metabolic profile characterized by markedly elevated HOMA index, Hb1Ac levels, and hepatic enzymes GGT and ALT across the tertiles. Conversely, there was a significant reduction in HDL cholesterol levels within these tertiles. When patients were stratified according to their subcutaneous fat content, we found increased CRP levels and lower CTx serum concentration across the SFA tertiles. Lastly, patient stratification according to the SFA/VFA ratio showed the most significant changes among tertiles. Subjects in the upper tertile presented significantly higher levels of CRP and HDL, while HOMA index, hemoglobin, CTx, triglycerides, and GGT were reduced (Fig. 2). We also explored the association of SFA/VFA ratio with cardiometabolic traits in sex-adjusted logistic models. One unit increase in the SFA/VFA ratio was associated with reduced odds ratios (ORs) for dyslipidemia (OR:0.370, CI95%: 0.125–0.816, *p* = 0.038), high blood pressure (OR: 0.321, CI95%: 0.126–0.652, *p* = 0.007), and type 2 diabetes (OR: 0.286, CI95%: 0.084–0.710, *p* = 0.022). These results prove that the SFA/VFA ratio is an excellent metric to differentiate the metabolic status of individuals with similar perceived obesity (same BMI and same waist circumference in the three tertiles).

### Identification of genes differentially expressed in subcutaneous adipose tissue according to the SFA/VFA ratio

**Fig. 3:**
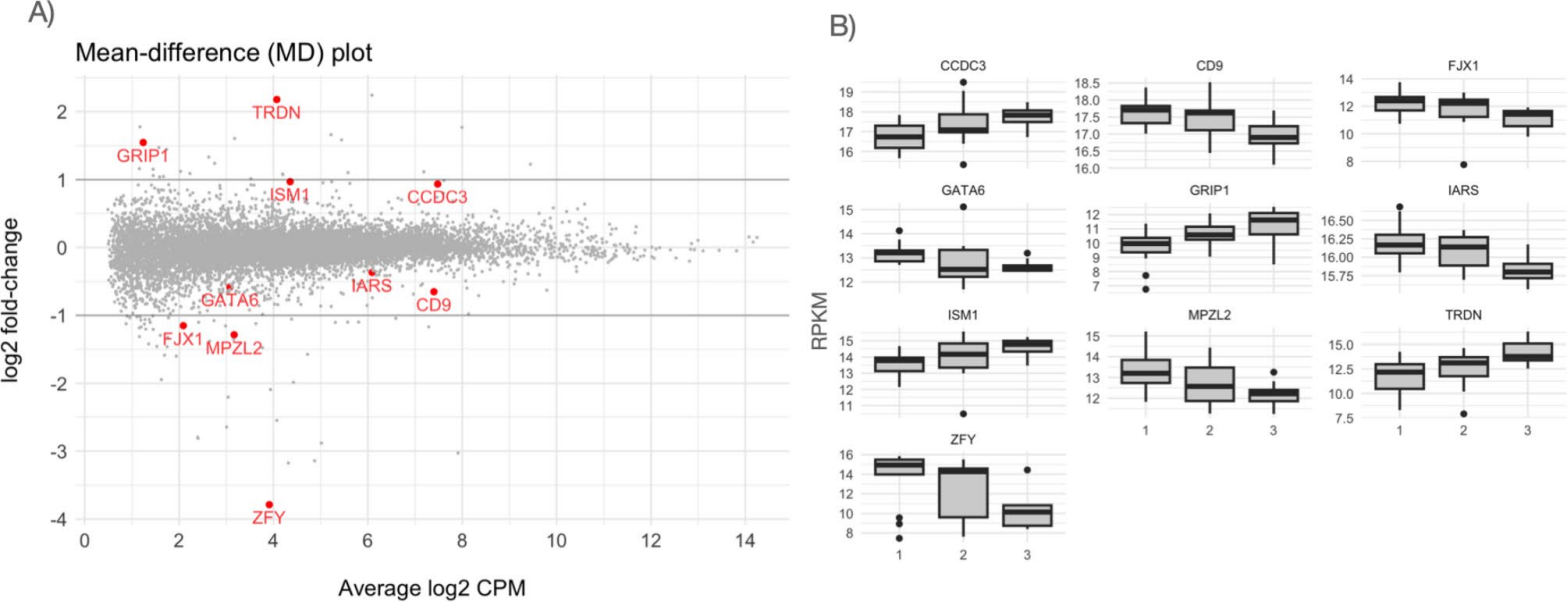
Genes differentially expressed in scWAT according to the SFA/VFA ratio. (**A**) MD plot showing the log-fold change and average abundance of each gene. Significantly (FDR ≤ 0.1) up and down DE genes are highlighted in red. (**B**) Variation of differentially expressed genes across tertiles of the SFA/VFA ratio. RPKM: Reads Per Kilobase per Million mapped reads

To identify potential biomarkers that reflect the abdominal fat partitioning, we carried out a global gene expression analysis in scWAT biopsies from 45 donors using edgeR [12] and contrasting the lower tertile (T1) vs. the upper tertile (T3) of de SFA/SVA ratio. Ten differential expressed genes (DEGs) with false discovery rate (FDR) < 0.1 were identified (Fig. 3A). Four of them (*CCDC3*, *GRIP1*, *ISM1*, and *TRDN*) showed a positive association, increasing their expression as SFA/VFA ratio increased, while six of them (*CD9, FJX1, GATA6, IARS, MPZL2*, and *ZFY*) showed a negative association (Fig. 3B).

Among those DEGs, *CCDC3 and ISM1* were genes encoding proteins previously reported to be secreted by adipose tissue. As our final goal was to find a non-invasive biomarker, these candidate genes were selected for subsequent analysis.

### Validation of adipose tissue expression and serum levels of CCDC3 and ISM1

**Fig. 4:**
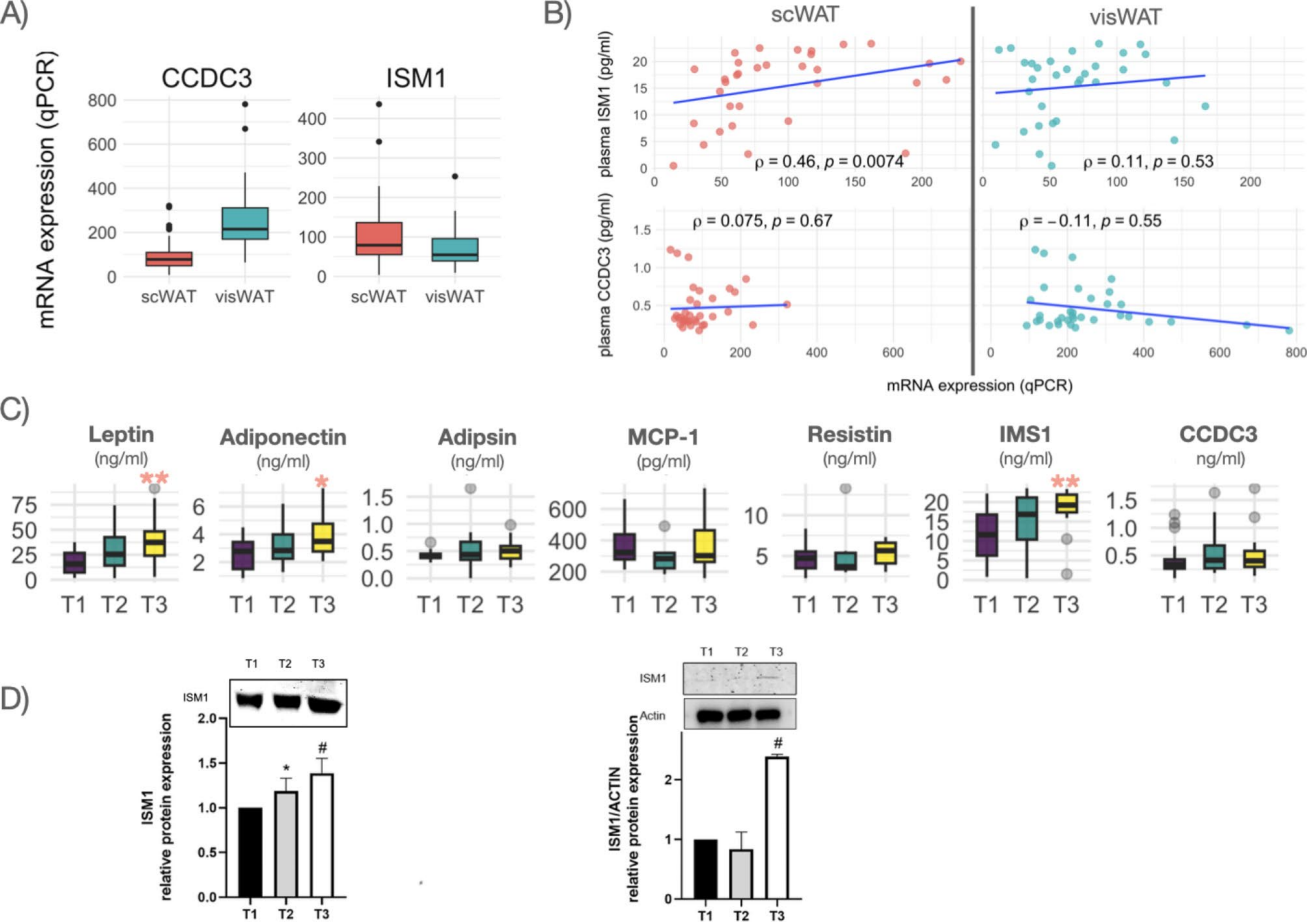
Expression of CCD3 and ISM1 by qPCR in the subcutaneous and visceral adipose depots and association of the SFA/VFA ratio with serum adipokines. (**A**) mRNA expression of CCDC3 and ISM1 in subcutaneous (scWAT) and visceral (visWAT) adipose tissues. (**B**) Depot specific correlation of serum CCD3 and ISM1 with mRNA expression levels. (**C**) Serum levels of different adipokines in individual with obesity, stratified by the SFA/VFA ratio. p for trend across tertiles: *<0.05, **<0.01

mRNA levels of *CCDC3* and *ISM1* were measured by qPCR in scWAT and visWAT. We found a high correlation between qPCR- and ampliseq-measured expression levels ( r = 0.83 and r = 0.75 for CCDC3 and ISM1, respectively, both *p* < 0.001). We also found a depot-specific expression in which *ISM1* mRNA levels were significantly higher in scWAT (*p* = 0.015), while *CCDC3* was more expressed in visWAT (*p* < 0.001)(Fig. 4A).

Next, we investigated the correlation of plasma values of ISM1 and CCDC3 measured by ELISA and their expression values in scWAT and visWAT. Interestingly, this correlation was observed only for ISM1 in scWAT (r = 0.46, *p* = 0.007) but not in visWAT (Fig. 4B). Conversely, CCDC3 plasma levels did not correlate with the expression levels in either scWAT or visWAT.

### Association of the SFA/VFA ratio with serum adipokines

Finally, we measured the serum levels of well-characterized adipokines and correlated these values with the SFA/VFA ratio. We did not observe an association of adipsin, MCP-1, or resistin, but a significant increase across SFA/VFA ratio tertiles was observed for leptin and adiponectin (Fig. 4C).

Regarding the newly identified adipokines, we did not observe an association of CCDC3 with the SFA/VFA, which aligns with the lack of association between scWAT expression and serum levels. However, a significant increase across SFA/VFA ratio tertiles was observed for ISM1. This upward trend in ISM1 was further validated through Western blot analysis in both plasma and scWAT (Fig. 4D), confirming the potential of this adipokine as a biomarker of adipose tissue distribution.

### Biomarker evaluation and insights into ISM1’s metabolic role

**Fig. 5:**
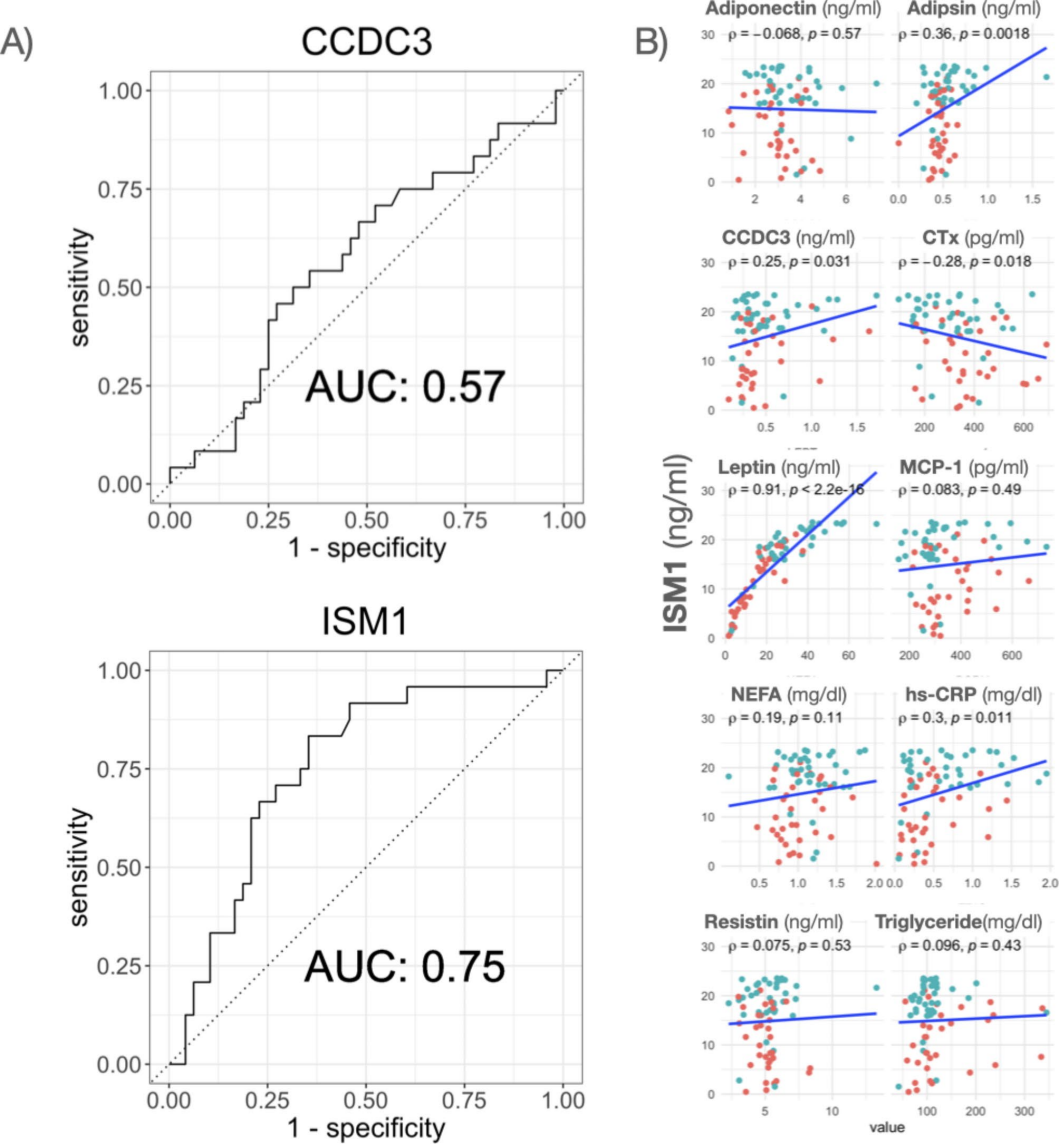
Sensitivity and specificity of CCDC3 and ISM1 as biomarkers of elevated SFA/VFA ratio and correlation analysis of ISM1 and other metabolic paramaters. (**A**) Receiver operator curves (ROC) for prediction of being in the first tertile (T1) of the SFA/VFA ratio. (**B**) Correlations between serum ISM1 and other metabolic parameters

We conducted a Receiver Operating Characteristic (ROC) analysis to assess the efficacy of ISM1 and CCDC3 as biomarkers in distinguishing subjects within the upper tertile of the SFA/VFA ratio, representing individuals with the most favorable metabolic profile. Our analysis found that CCDC3 had an Area Under the ROC Curve (AUC) value of 0.57, indicating that it performed relatively poorly as a predictor (Fig. 5).

However, ISM1 exhibited a significantly higher AUC of 0.75, demonstrating a fair ability to discriminate between individuals in the upper tertile of the SFA/VFA ratio and those outside this category, underscoring its potential clinical relevance and utility in the stratification of obese subjects with superior metabolic profiles based on adipose tissue distribution.

Lastly, we performed a correlation analysis among plasma ISM1 and other metabolic parameters to obtain some clues about the potential role of ISM1 in metabolic regulation in obesity. We found significant correlations with several metabolic markers. Notably, it displayed strong associations with leptin and PCR. Additionally, ISM1 showed correlations with adipsin and CTx, both linked to glucose metabolism, indicating possible involvement in glucose regulation pathways. In contrast, no significant correlations were observed between ISM1 and adiponectin, NEFA, resistin, or plasma triglycerides. These findings imply that ISM1 may play multifaceted roles in metabolic regulation, warranting further investigation into its specific mechanisms and functions within these metabolic pathways.

## Discussion

The primary aim of this study was to discover a non-invasive biomarker to predict the distribution of abdominal adipose tissue in individuals with obesity, and consequently, to assess the metabolic risks linked to fat mass accumulation. To achieve this goal, we conducted a transcriptomic analysis on scWAT biopsies to identify candidate genes associated with the SFA/VFA ratio. Subsequently, we assessed their levels in serum. We unveiled a novel adipokine, ISM1, whose serum levels correlated with the SFA/VFA ratio. These findings suggest that ISM1 could predict abdominal fat partitioning and be a potential biomarker for evaluating obesity-related health risks.

Obesity increases the risk of developing metabolic disorders, such as cardiovascular disease, type 2 diabetes, hypertension, or fatty liver [14, 15]. However, not all forms of obesity are equally dangerous: despite higher percentages of body fat, some individuals are at less risk for certain chronic obesity-related complications [4, 6]. Data suggest that fat’s physical location dramatically influences disease risk [5, 7]. Accumulation of adipose tissue in the upper body, in the abdominal region (android obesity), is associated with the development of obesity-related comorbidities and even all-cause mortality [16]. In contrast, population studies have shown that fat accumulation in the lower body, in the gluteal-femoral region (gynoid obesity), is associated with protective lipid and glucose profiles and decreased cardiovascular and metabolic disease prevalence [5–7].

However, the situation becomes more intricate when considering that the association of abdominal obesity with metabolic risk is primarily observed when subcutaneous white adipose tissue (scWAT) reaches its maximum storage capacity and fails to handle lipid storage adequately. This results in the redirection of lipid flux to visceral white adipose tissue (visWAT), leading to the accumulation of ectopic fat. Such a process triggers insulin resistance through mechanisms involving lipotoxicity and inflammations [17], as outlined in the adipose tissue expandability hypothesis [18]). Therefore, it is critical to evaluate not only the quantity and the location of fat in the body but also to distinguish between visceral and subcutaneous abdominal depots. Considering that, the first objective of our work was to verify whether the scWAT/visWAT ratio was suitable to stratify patients with obesity according to their metabolic risk [19, 20].

Several studies have lend some support to this theory, demonstrating that in obesity, the regional distribution of adipose tissue strongly correlates with several important metabolic variables [3]. It has been broadly reported that VFA, but not total adiposity or SFA, is associated with glucose intolerance, suggesting an insulin-resistant state [21, 22]. In addition to being associated with disturbances in insulin-glucose homeostasis, visceral obesity has been related to alterations in plasma lipoprotein-lipid levels, particularly increased plasma triglyceride and low HDL concentrations [23, 24], as well as with liver disease [25].

Our results align with these previous data, being the stratification according to the SFA/VFA ratio the one that showed the major metabolic differences between tertiles. Patients in the upper tertile of SFA/VFA ratio (more subcutaneous and less visceral fat) had similar BMI and waist circumference than those in the lower tertile. However, they presented lower levels of indicators of metabolic risk, with better carbohydrate metabolism (reduced HOMA as well as Hb1Ac and CTx levels), better lipid profile (lower triglycerides and higher HDL serum concentration), and decreased levels of liver damage markers (GGT and ALT). This suggests that despite similar antropometric measurements (BMI, waist circumference), body fat distribution was linked metabolic disease severity markers, as expected.

Paradoxically, we found that hs-CRP levels increased with subcutaneous expansion (subcutaneous obesity model or upper SFA/VFA ratio). CRP is the most frequently employed marker for acute-phase reactants associated with inflammation [26]. Previous studies had reported a positive association between CRP and visceral fat [4, 27]. The association observed with scWAT in our cohort could be attributed to the augmented size (hypertrophy) of adipocytes in the subcutaneous depot [28, 29], leading us to hypothesize that heightened hs-CRP levels may potentially indicate the threshold of adipose expansion in our patients with morbid obesity.

Considering previous data and our results, the SFA/VFA ratio is a good indicator of metabolic risk. Nevertheless, the challenge lies in implementing this assessment, as advanced techniques like body composition analysis and imaging are often complex and impractical in many clinical settings. Therefore, our second aim was to identify a non-invasive biomarker that accurately reflects adipose tissue distribution.

Adipokines have been broadly proposed as non-invasive biomarkers of obesity, as their production is often dysregulated in obese individuals and contributes to the pathogenesis of obesity-associated metabolic complications [30, 31]. For instance, circulating adiponectin levels have been inversely correlated with body weight, visceral fat accumulation, and metabolic disease risk [32, 33]. At the same time, a positive association has been reported between leptin levels and the percentage of fat mass [34]. This evidence is also observed in our cohort, where adiponectin levels decreased as visceral fat decreased and leptin levels increased as total subcutaneous fat mass increased. Since the synthesis of adipokines reflects adipose tissue function in overall metabolic homeostasis, we assumed that perhaps differences in the expression of not-yet known adipokines produced by the scWAT could reveal divergence in the metabolic phenotype. Therefore, we performed an RNAseq analysis in scWAT, and we identified two genes whose expression increased as the SFA/VFA ratio increased and which were predicted to encode adipose tissue-secreted proteins; CCDC3 and ISM1.

CCDC3 is a secretory protein reported to be highly expressed in adipose tissue, regulated by insulin and pioglitazone, and suppressed by TNF-alpha, isoproterenol, and norepinephrine [35]. Its hormone-like role in regulating lipid metabolism has been described in an autocrine manner, regulating adipocyte lipogenesis [36], and in a paracrine manner, regulating liver lipid metabolism [37]. Moreover, it has been linked to obesity, especially visceral fat accumulation [38]. Our results showed a positive association between CCDC3 expression in scWT and the SFA/VFA ratio. However, this correlation was not replicated in serum CCDC3 levels, which excludes this adipokine as a valid biomarker of scWAT functionality. CCDC3 expression levels were higher in visceral instead of subcutaneous fat, and there could be different mechanisms regulating its expression and secretion in both fat depots.

The second potential biomarker was ISM1, a secreted protein initially discovered in fetal brain development and expressed in the vasculature, skin, immune cells, and lungs [reviewed in [39]]. ISM1 has been recently identified in mouse and human adipocytes as an adipokine that has essential metabolic roles in multiple tissues, promoting glucose uptake, inhibiting hepatic lipogenesis [40, 41], and stimulating protein synthesis [42], thus improving hyperglycemia and reducing lipid accumulation in mouse models. Moreover, ISM1 expression in adipocytes and circulating ISM1 levels have been associated with obesity and reduced risk of type 2 diabetes mellitus (T2DM) [43, 44]. However, nothing is known about its association with adipose tissue distribution.

Our data showed that ISM1 mRNA expression levels scWAT were strongly associated with the SFA/VFA ratio. However, no such association was observed in visWAT. In the same vein, circulating ISM1 levels were positively associated with SFA and the SFA/VFA ratio but not with VFA. This suggests that the elevated ISM1 levels observed could potentially originate from subcutaneous adipose cells and exert endocrine influences on other tissues. We posit that that ISM1 secreted by scWAT has a beneficial role in whole-body energy homeostasis, which aligns with the protective function previously reported for this adipokine [41].

In our study, we conducted a comprehensive correlation analysis to shed light on the metabolic regulation of ISM1. We found significant correlations between ISM1 and CCDC3 and hs-PCR, the later could be indicative of the presence of an exhausted scWAT adipocytes reaching their limit of expansion. Notably, ISM1 was also associated with adipsin and CTx, both linked to glucose metabolism [45, 46], hinting at a role of this new adipokine in glucose regulation. Interestingly, we found ISM1 plasma levels highly correlated with leptin levels. Leptin is a well-known adipokine mainly produced by scWAT in proportion to the amount of fat mass and is involved in regulating food intake and glucose and lipid metabolism, among others [30]. Although leptin is a well-characterized adipokine, studies associating this hormone with ISM1 are scarce. Recent evidence revealed a simultaneous increase in the expression of ISM1 and leptin in the adipose tissue of mice fed high fat diet [41]. At the same time, no significant associations were identified between serum levels of these two adipokines in pre-puberal boys [44]. This association warrants further investigation as a common regulatory mechanism for both adipokines could exist. Both ISM1 and leptin seem to be expressed mainly by scWAT, increasing their levels in obesity. In addition, both seem to have a role in regulating glucose metabolism, so that future research could clarify the role of the combined function of these adipokines in the maintenance of energy homeostasis.

These findings collectively underscore ISM1’s potential involvement in various metabolic processes, laying the foundation for future research into its precise metabolic functions. However, important questions also arise from our current findings: the molecular mechanism regulating ISM1 expression in scWAT and its association with leptin, or the target tissues and function of this adipokine. Besides, our results mainly concern individuals with obesity, and how this protein is expressed and secreted in non-obese also warrants further investigations. Moreover, further validation in an independent cohort must be performed before proving its potential as a biomarker of adipose tissue distribution. In the same vein, longitudinal investigations would help elucidate whether alterations in an individual’s fat distribution following bariatric surgery can result in corresponding changes in their serum ISM1 levels.

## Conclusions

Our research proves the importance of distinguishing adipose tissue location to classify obesity phenotypes. It proposes ISM1 as a novel non-invasive biomarker to predict abdominal fat partitioning and, consequently, to evaluate obesity-related health risks.

## Data Availability

The datasets used and/or analyzed during the current study are available from the corresponding author on reasonable request.
